# Towards selective electrochemical conversion of glycerol to 1,3-propanediol†

**DOI:** 10.1039/c8ra00711j

**Published:** 2018-03-19

**Authors:** Olusola O. James, Waldemar Sauter, Uwe Schröder

**Affiliations:** Institute of Environmental and Sustainable Chemistry, Technical University Hangenring 30 38106 Braunschweig Germany uwe.schroeder@tu-braunschweig.de; Chemistry Unit, Kwara State University Malete, P.M.B. 1530 Ilorin Nigeria olusola.james@kwasu.edu.ng

## Abstract

1,3-propanediol (1,3-PD) is a bulk chemical with myriad applications in polymers, lubricants, cosmetics, foods industries and in the synthesis of heterocyclic compounds. Current commercial production of 1,3-PD involves a thermocatalytic process using acrolein (DuPont) and ethylene oxide (Shell) as starting feedstock. These feedstocks are petroleum-based and there are many efforts at using glycerol as low cost biomass-derived feedstock for 1,3-PD production. A number of catalyst designs and bacterial & fungal strains are being explored for respective catalytic and fermentation routes to glycerol-to-1,3-PD. However, the electrochemical method received little attention for the purpose. In this work, in order to explore the possibility of using partly refined glycerol byproduct of biodiesel production as feedstock, we investigated conversion and 1,3-PD selectivity of glycerol electrolysis in chloride media. We demonstrated selective glycerol-to-1,3-PD conversion using Pt or RuO_2_-based dsa as anode and Zn or Pb as cathode in NaCl and KCl at pH 1. This electrochemical glycerol-to-1,3-PD conversion is not only green, it is a potential process network loop between biodiesel production and chlor-alkali industry.

## Abstract 2

Glycerol electrolysis in chloride media allows a green and sustainable route production to 1,3-propanediol and also benefits from easy integration into the chlor-alkali industry.

## Graphical abstract

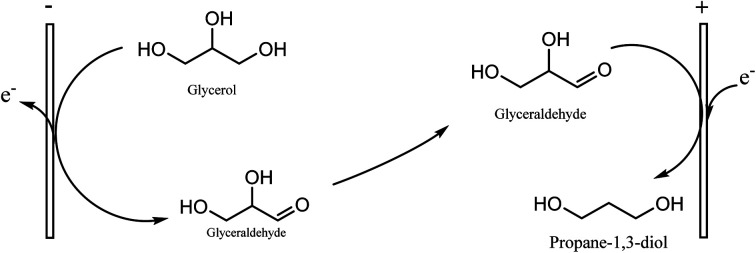

## 1 Introduction

Despite the current low price of petroleum, non-oil producing nations have concerns about the long term security of the petroleum supply.^1–3^ Moreover, climate and global warming remain key drivers to replace the current petroleum based transportation and organic chemical industries with renewable resources.^4,5^ Presently, the entire transportation sector depends almost exclusively on petroleum. Also, petroleum provides the feedstocks and the energy inputs for the petrochemical industry for production of basic and fine organic chemicals. The economy of the petrochemical industry is built around the high-energy density, abundance and low cost of petroleum. These set of attributes present formidable economic challenges for adoption of biofuels and renewable substitutes to petroleum in the transportation sector. However, conversion of biomass-derived feedstocks into high value commodity chemicals is gaining interest as a more plausible path to reduce dependence on petroleum.^6–9^ And where possible, use of renewable energy input for biomass conversion will be an added step towards waning reliance on fossil fuels.

Glycerol has been identified as an important biomass-derived feedstock to a variety of commodity chemicals.^10,11^ It is readily available as a byproduct of the oleochemical industry and enjoys wide application as moisturizer in cosmetic products. Increasing biodiesel productions in recent times has brought about an oversupply of glycerol for its traditional applications.^12,13^ Hence, there are efforts at channeling the oversupply of glycerol towards serving as feedstock to valuable C_3_ chemicals. Examples of derivable high value C_3_ chemicals from glycerol include: propylene glycols, hydroxyacids, and diacid.^14–16^ Among the possible glycerol derivable C_3_ chemicals, 1,3-propanediol (1,3-PD) is of special interest because it is a bulk chemical that has wide applications in polymers, lubricants, cosmetics, foods and in the synthesis of heterocyclic compounds.^17^

The traditional routes of commercial synthesis of 1,3-PD use acrolein (DuPont) and ethylene oxide (Shell) as starting feedstock. The DuPont route involves hydration of acrolein to 3-hydroxypropionaldehyde (3-HPA), followed by hydrogenation of 3-HPA to produce 1,3-PD. While shells route involves hydroformylation of ethylene oxide to 3-hydroxypropanal and subsequent hydrogenation to 1,3-PD.^18^ These traditional commercial routes to 1,3-PD depend exclusively on petroleum-derived feedstocks. DuPont and Tate & Lyle developed and commercialized a glucose-based process route to 1,3-PD. The process involves fermentation of corn syrup using genetically modified *e. coli* with addition of vitamin B_12_. This bioconversion route to 1,3-PD is reported to consume 40% less energy than the traditional petroleum-based route. However, the need for vitamin B_12_ addition has an impact on the economics of the glucose-based process route to 1,3-PD.^19^ Moreover, this bio-process route to 1,3-PD involved glycerol as an intermediate, which further motivates interest in glycerol to 1,3-PD considering oversupply of glycerol from biodiesel production.

Although the glycerol-to-1,3-PD fermentation process is being explored, the productivity so far obtained is lower compared to the glucose-based fermentation. In recent times there are a number reports on heterogeneous catalytic hydrogenolysis of glycerol to 1,3-propanediol (1,3-PD).^20–22^ This route requires high temperature and pressure conditions and hydrogen supply which at present is most economically produced *via* steam reforming of natural gas or a petroleum feedstock. In this study, we examined electrochemical conversion of glycerol to 1,3-PD. Since 1,3-PD is a bulk chemical with many applications, electrochemical glycerol-to-1,3-PD conversion is not only a green and sustainable route to 1,3-PD but also it can serve as a means of storing excess renewable electricity.

## 2 Experimentals

### 2.1 Chemicals

All chemicals used in this study were of analytical grade. For qualitative and quantitative analysis, reference materials and solvents were used as purchased, without purification. Glycerol, dihydroxyacetone, glyceric acid, glyceraldehyde, acetol, 1,3-propanediol, acetone, 2-propanol and 1-propanol.

### 2.2 Electrode materials

AgCl, sat. KCl electrode was used (SE11, Sensortechnik Meinsberg, Germany), 0.197 *vs.* standard hydrogen electrode (SHE) as reference. All used electrodes are listed in Table 1 with their respective purity and surface area.

**Table 1:** Purity and properties of the electrodes used

Electrode material	Purity	Electrode surface (cm^2^)
Lead (Pb)^a^	99.999	11
Zinc (Zn)^b^	99.99	11
Platinum	99.9	11
Ruthenium based dimensional stable anode (RUA)^c^		11

a ChemPUR, Germany.

b Umicore Galvanotechnik GmbH, Germany.

c Ti Anode Fabricators Pvt. Ltd, Chennai – 126, India.

### 2.3 Electrochemical procedure

All electrochemical reactions were conducted under potentiostatic control using a potentiostat/galvanostat SP50 (Bio-Logic SAS, Claix, Frankreich). All experiments used a three-electrode configuration and were stirred continuously with a magnetic stirrer. The undivided cell is a single 40 ml chamber cylindrical glass cell with a 1 cm neck window fitted with a Teflon cork stopper bearing and the anode and cathode terminals are 2 cm apart. The divided cell is a 40 ml each, two-chamber H-type glass cell. The anode and cathode terminals are 10 cm apart, the anode and the cathode chambers were separated *via* a cation exchange membrane (fumasep FKE, Fumatech, Germany).

### 2.4 Analysis

Quantitative analyses were performed by high performance liquid chromatography (HPLC), by means of a refractive index (RI) detector (Spectrasystem P4000, Finnigan Surveyor RI Plus Detector, Fischer Scientific) equipped with HyperREZ XP carbohydrate H + 8 μm (S/N: 026/H/012-227) column. Sulphuric acid (0.005 N, flow rate 0.5 ml min^−1^) was used as eluent. The column was operated at different temperature depending on sample composition, the refractory index detector was operated at 42 °C. Glycerol conversions and products selectivities are calculated as follows:
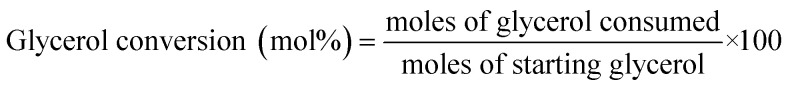

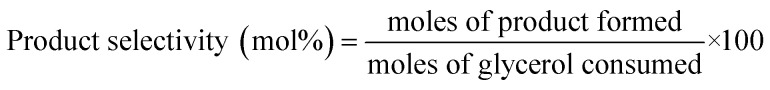


## 3 Results and discussion

### 3.1 Electrode selectivity requirements

**Scheme 1:**
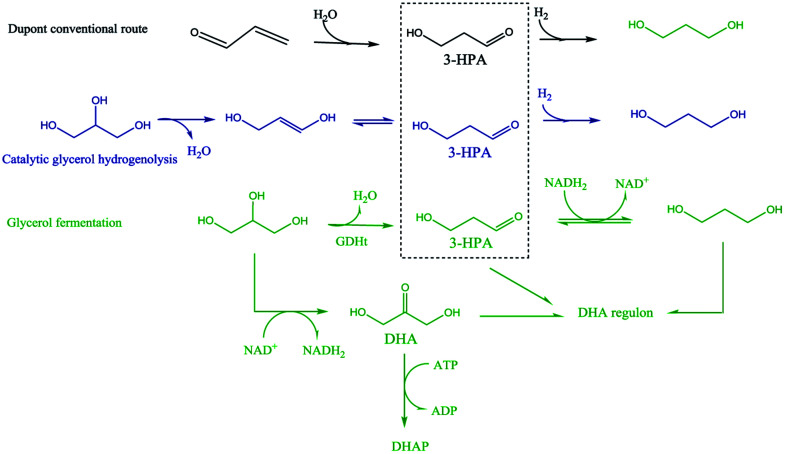
Mechanistic representation of reaction route to 1,3-Propanediol *via* glycerol fermentation, catalytic glycerol hydrogenolysis, and conventional Dupont commercial synthesis of 1,3-PD from acrolein.

It is interesting to note that 3-hydroxypropionaldehyde (3-HPA) is a common intermediate in 1,3-PD production by conventional Dupont commercial synthesis of 1,3-PD from acrolein, glycerol metabolism (fermentation) and heterogeneous catalytic glycerol hydrogenolysis (Scheme 1). The fermentation and the catalytic hydrogenolysis conversion routes followed the same tandem dehydration and hydrogenation steps to 1,3-PD. This common feature in the routes seems to be an indication of 3-HPA as a critical intermediate for glycerol to 1,3-PD conversions, and the electrochemical method may be no exception. Thermocatalytic hydrogenolysis of glycerol often involves tandem dehydration and hydrogenation. These thermochemical reaction steps are difficult to replicate electrochemically, especially the dehydration step. Hence direct electro-hydrogenolysis of glycerol appears electrochemically inaccessible. Moreover, as shown in the reaction mechanism of the fermentation method, dihydroxyacetone (DHA) intermediate could constitute a drain on 1,3-PD productivity in an electrochemical method. Thus, none of the routes in Scheme 1 is replicable for achieving electrochemical conversion of glycerol to 1,3-PD.

The Clemmensen reduction of carbonyl groups is well documented in organic chemistry for achieving conversion of a carbonyl group to a methylene bridge.^23^ In the conventional Clemmensen reduction, zinc is typically used as a stoichiometric reductant. However, in cathodic Clemmensen-type reduction electrons are the reductant while zinc plays a catalytic role. The cathodic Clemmensen-type reduction has been reported for other metallic (lead, cadmium and mercury) electrodes;^24–27^ and it has been exploited for biomass-to-biofuel production.^28,29^ Thus, it is a potent electrochemical step for achieving a conversion of a carbonyl group to a methylene group. So, tandem selective oxidation of glycerol to DHA, and Clemmensen-type cathodic reduction of DHA seems a plausible reaction route for achieving glycerol-to-1,3-PD electrochemical conversion. However, there are other pathways that compete with Clemmensen mechanism in cathodic reductions of alpha-hydroxycarbonyl compounds. Carbonyl-to-hydroxyl or dehydroxylation of alpha-hydroxyl group(s) to a carbonyl group had been identified as competing pathways to Clemmensen mechanism in cathodic reductions of alpha-hydroxycarbonyl compounds.^30,31^ Preliminary studies using Clemmensen reduction active metal electrodes (Pb and Zn) in acidic media (HCl, H_2_SO_4_ and H_3_PO_4_) showed that electroreduction of DHA followed the dehydroxylation path. On the other hand, the dehydroxylation path favours electroreduction of glyceraldehyde to 1,3-PD in neutral and acidic pH. This is in line with deduction from the fermentation route that a selective electrochemical glycerol-to-1,3-PD process should avoid DHA as an intermediate. Therefore, selective anodic oxidation of glycerol to glyceraldehyde is critical to achieve high 1,3-PD productivity.

The nature of electrolyte is an important variable in electrochemical conversions. Anodic oxidation can *via* direct electron transfer from the substrate to the anode or indirectly through an anodically generated oxidant species. Regardless of the mechanism of the anodic oxidation, the main target at the anode is to achieve selective glycerol-to glyceraldehyde oxidation. Glycerol is made up of two primary and one secondary hydroxyl groups. Selective glycerol to glyceraldehyde oxidation will entails preferential oxidation one of the primary hydroxyl groups of glycerol over its secondary hydroxyl group. Preferential chemical oxidation of a primary hydroxyl group over a secondary hydroxyl group had been reported using aqueous hypochlorite as oxidant and 2,2,6,6-tetramethylpiperidinyl-1-oxy (TEMPO), catalyst.^32^ And using appropriate anode hypochlorite and other aqueous chlorine species can be anodically generated. Moreover, the use of chloride media has the potential of creating a network loop of electrochemical chlor-alkali cell, glycerol production (from biodiesel production) and electrochemical glycerol-to-1,3-PD conversion. Thus, for simplicity, we are inclined to explore carrying out the glycerol oxidation in chloride media without the complication of a TEMPO catalyst. Combination of glyceraldehyde selectivity and stability in chloride media requirements place stringent criteria on selection of the anode for achieving the electrochemical conversion. Potential candidates that can meet the selectivity requirement are platinum, gold and ruthenium based dimensionally stable anode (RuO_2_-dsa). The Gold anode is prone to corrosion in chloride media and platinum is expensive and may be cost prohibitive for large scale application. RuO_2_-dsa is already an established anode in the chlor-alkali industry.^33,34^ In chloride media, ruthenium oxide-based dsa is selective to chlorine formation and aqueous chlorine species are an potential oxidant for glycerol oxidation. Hence, RuO_2_-dsa was investigated first to ascertain anodic glyceraldehyde selectivity in our quest towards selective electrochemical conversion of glycerol to 1,3-PD.

### 3.2 Glycerol electrolysis using RuO_2_-dsa anode and Pb & Zn cathode

**Fig. 1:**
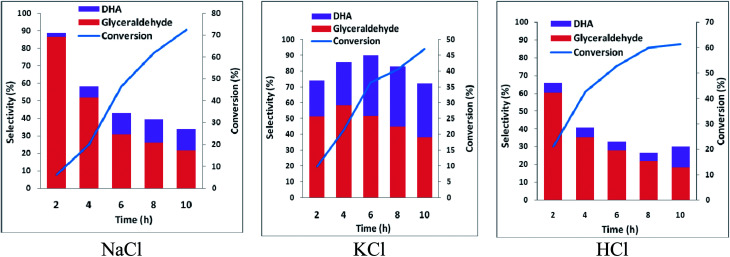
Conversion and selectivity of oxidation of 0.25 M glycerol in a divided cell using Ti–RuO_2_ working electrode and Pt counter electrode in 0.5 M chloride solutions (potential 2.5 V *vs.* AgCl/Cl sat. KCl, temperature 25 °C).

**Fig. 2:**
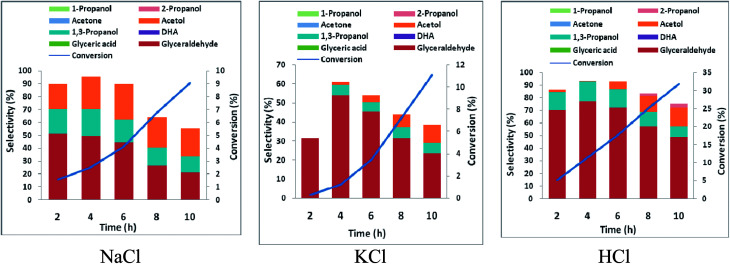
Conversion and selectivity of oxidation of 0.25 M glycerol in an undivided cell using Pb working electrode and Ti–RuO_2_ counter electrode in 0.5 M chloride solutions (potential −1.8 V *vs.* AgCl/Cl sat. KCl, temperature 25 °C).

**Fig. 3:**
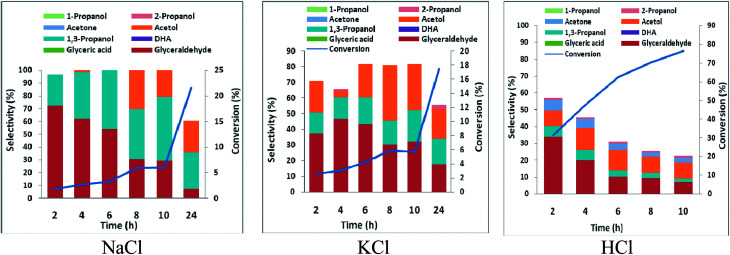
Conversion and selectivity of oxidation of 0.25 M glycerol in a undivided cell using Zn working electrode and Ti–RuO_2_ counter electrode in 0.5 M chloride solutions (potential 2.5 V *vs.* AgCl/Cl sat. KCl, temperature 25 °C).

**Fig. 4:**
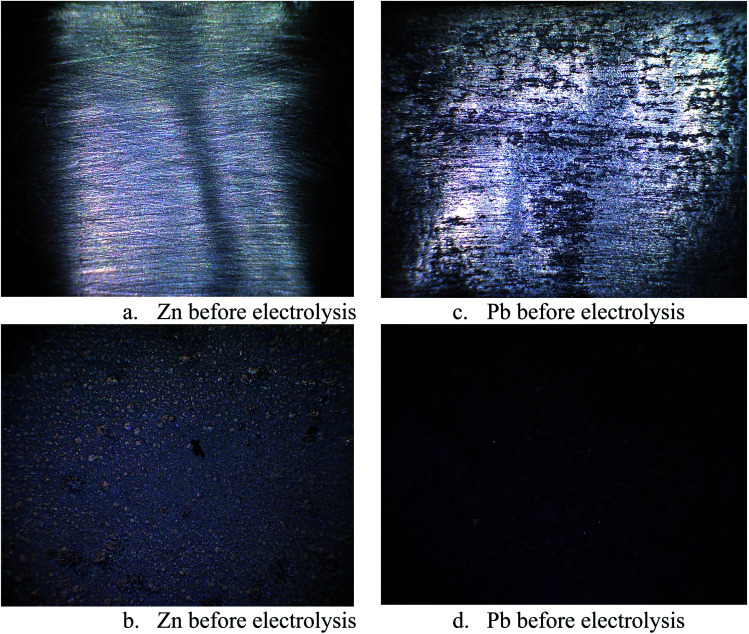
Confocal micrograph image of the surface of the Zn and Pb as working electrode before and after electrolysis 0.25 M glycerol in an undivided cell with Ti–RuO_2_ counter electrode in 0.5 M HCl solution (potential −1.8 V sat'd AgCl/Cl, Temperature 25 °C).

In order to ascertain anodic glyceraldehyde selectivity we investigated the oxidation of glycerol in chloride media using RuO_2_-dsa anode. Fig. 1 shows glycerol conversions and product selectivities in a divided cell. Although the ideal anodic result is 100% glyceraldehyde specificity, the results obtained are less than the ideal glyceraldehyde selectivities. The glyceraldehyde selectivities obtained are sufficiently high enough to feed the coupled cathodic dehydroxylation to 1,3-PD. The results of the tandem glycerol oxidation and cathodic dehydroxylation in chloride media over Pb and Zn electrodes are presented in Fig. 2 and 3 respectively. The neutral media displayed lower glycerol conversions compared to the HCl medium. Also 1,3-PD selectivity is higher in NaCl than in KCl medium. While higher glycerol conversions were obtained in HCl medium, the Zn cathode *vs.* RuO_2_-dsa anode gave higher glycerol conversion than the Pb cathode *vs.* RuO_2_-dsa anode. The detailed reason for this observation is not clear at the moment. However, it is observed that the Zn electrode was corroded as indicated by about 8% mass loss and a high zinc concentration was found in the reaction cell content after the electrolysis. Although mass gain of 0.15% was observed for the Pb electrode, ICP-OES analysis of the reaction cell content also indicates corrosion of the Pb electrode (Tables S1a and b†). As shown in Fig. 4 increased surface roughness was observed for the Zn electrode while that of Pb became smoother after the electrolysis. This is an indication of surface reconstruction during the course of the cathode half reaction. The increased roughness of the Zn electrode after the electrolysis suggests dissolution and re-deposition of Zn as dendrite-like micro-crystals. Although dissolution of Pb and Zn in acidic media is thermodynamic downhill, moreover the chlorine oxidant species are corrosive to Pb and Zn. PbCl_2_ has a smaller solubility product constant than ZnCl_2_. So the Pb electrode benefits from protective surface coverage of PbCl_2_ which reduced the rate of Pb corrosion in the reaction environment.

The observed mass gain of Pb electrode suggests adsorption of edduct or counter ion (chloride ion) on the Pb electrode surface. Zn lacks similar surface protection and may become impractical as a cathode for a long time or large scale electrolysis in such a highly corrosive reaction environment. The cathodic redeposition restores the Pb electrode much more efficiently than the Zn electrodes. Although the corrosion reaction is a drain on the current efficiency of the glycerol electrolysis, it may be beneficial to renewal of the electrode surface which may account for the higher glycerol conversion obtained in the electrolysis using the Zn cathode.

**Scheme 2:**
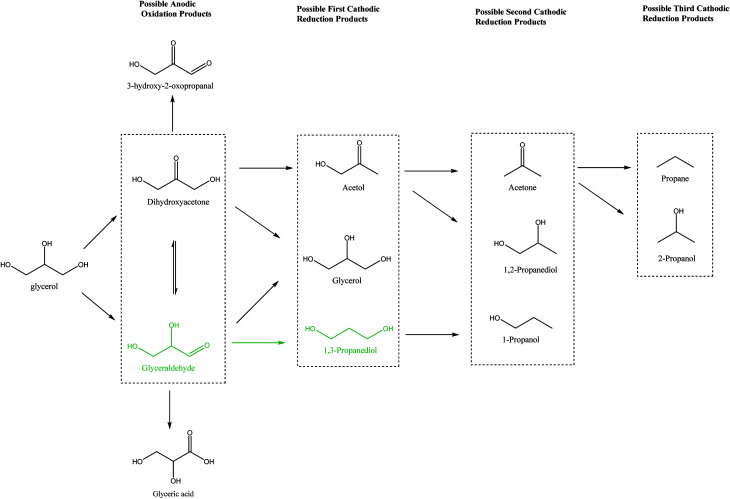
Possible pathways of tandem glycerol oxidation and cathodic reduction (Pb and Zn).

Purification of crude glycerol (from biodiesel synthesis or triglyceride saponification) usually involves an initial acidification to pH 1.^35^ Direct usage of the partially refined glycerol up to this stage will constitute a cheap feedstock for glycerol electrolysis to 1,3-PD. Encouraged by higher 1,3-PD productivity of Zn compared to Pb in neutral media, we tested the tolerance of Zn to pH 1. There was no significant difference in the mass of Zn plate electrode before and after 10 h of electrolysis. With reference to the neutral media glycerol conversion was unaffected by lowering the pH of the media to pH 1 using the Pb and the Zn electrode. However, with the Zn electrode, 1,3-PD selectivity was enhanced at pH 1. Similar enhancement of 1,3-PD productivity was not observed for Pb electrode (Fig. S3 and S4†). Despite initial high glyceraldehyde and low DHA selectivities of glycerol oxidation obtained in Fig. 1, selectivities of reduced products of DHA (acetol) and glyceraldehydes (1,3-PD) tend to equalize over the course of the electrolysis. This may be explained by a proposed pathway for the glycerol electrolysis depicted in Scheme 2.

### 3.3 Glycerol electrolysis using Pt anode *vs.* Pb and Zn cathode

Electrochemical conversion of glycerol has been a subject of many studies over the past three decades. Fuel cell application was the main driver of earlier studies on electrooxidation of glycerol. Large proportions of the studies were carried in alkaline media. Few of the studies were in acidic media. Generally, glycerol oxidation typically shows higher activity in alkaline media.^36^ Glycerate, dihydroxyacetone, and tartronate are reaction products over noble metals in alkaline media. The pathway of glycerol oxidation in alkaline media over a gold electrode had been exploited for electricity-chemical cogeneration.^37^ Selective electrochemical oxidation of glycerol at platinum has enjoyed focus of several theoretical and experimental reports. In acidic media, the pathway of glycerol oxidation over platinum surface is *via* the primary hydroxyl group, and modification of the platinum surface with Bi or Sb can tune the pathway towards the secondary hydroxyl group.^38,39^

It has been shown that electrooxidation of glycerol in acidic media over Pt is selective to glyceraldehyde at potentials below oxygen evolution.^40^ The glyceraldehyde selectivity was attributed to selective dehydrogenation of a primary hydroxyl group of glycerol oxidation *via* weakly adsorbed OH species on platinum and fast desorption of glyceraldehyde from Pt surface. This is a desirable selectivity for achieving the objective of selective glycerol-to-1,3-PD electrochemical conversion. However, glycerol conversions are very low at the potentials below oxygen evolution. High glycerol conversion is obtainable at potentials above oxygen evolution. But high glycerol conversion could be compromised with glyceraldehyde selectivity. This is because at potential above oxygen evolution, stable oxides form on the platinum surface. The oxides platinum surface increase glyceraldehyde adsorption on the electrode and promotes further oxidation to glyceric acid. This tendency nonetheless, for a head-to-head comparison with RuO_2_ dsa, we carried out glycerol oxidation in chloride media using platinum at 2.5 V *vs.* Ag/AgCl. Expectedly glyceric acid selectivities obtained were considerable; glycerol oxidation using platinum gave similar glyceraldehyde selectivities in neutral pH but higher glyceraldehyde selectivity in HCl compared to that obtained using RuO_2_ dsa. DHA selectivity was lower using platinum than RuO_2_ dsa, especially in KCl (Fig. S4†). For both platinum and RuO_2_ dsa, material balances of the reaction with time are attributable to undesired over oxidation of glycerol to CO_2_ and other side products that were not monitored.

**Fig. 5:**
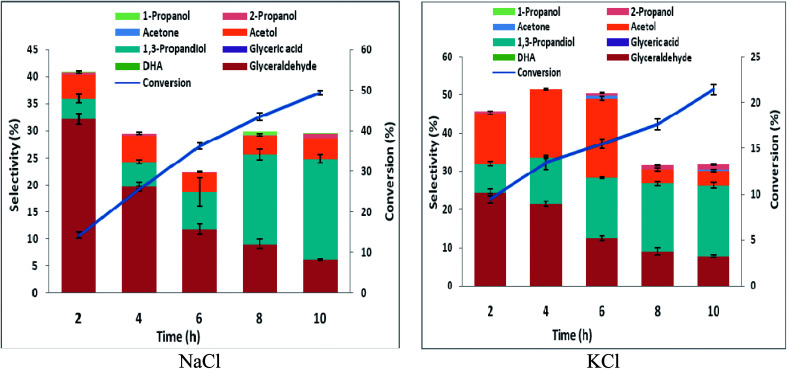
Conversion and selectivity of oxidation of 0.25 M glycerol in a undivided cell using Pb working electrode and Pt counter electrode in 0.5 M chloride solutions acidified with HCl to pH 1 (potential −1.8 V *vs.* AgCl/Cl sat. KCl, temperature 25 °C).

**Fig. 6:**
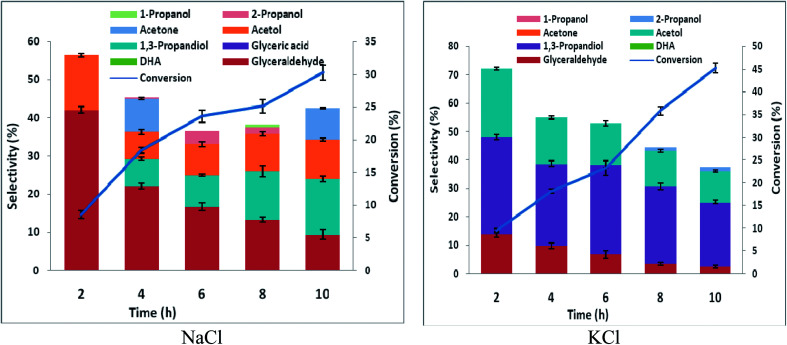
Conversion and selectivity of oxidation of 0.25 M glycerol in a undivided cell using Zn working electrode and Pt counter electrode in 0.5 M chloride solutions acidified with HCl to pH 1 (potential −1.8 V *vs.* AgCl/Cl sat. KCl, temperature 25 °C).

**Scheme 3:**
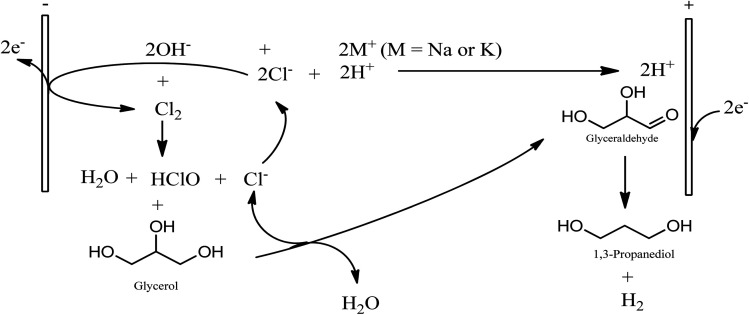
Proposed scheme of reaction steps of the glycerol electrolysis at pH 1 in chloride media.

The high glyceraldehyde selectivity obtained in electrooxidation of glycerol in HCl using platinum did not translate into expected high 1,3-PD selectivity in the corresponding glycerol electrolysis when platinum (as anode) was coupled with lead or zinc (as cathode). Notably, the electrolysis in HCl gave very low 1,3-PD selectivity and glycerol conversion was lowest using zinc (Fig. S5 and S6†). As explained in Section 3.2 with RuO_2_ dsa, zinc is subject to severe corrosion in HCl. However, platinum is less selective to chloride oxidation due to competitive adsorption between chloride and glycerol on the platinum surface. The amount of chlorine species produce during the electrolysis in HCl is lower using platinum than with RuO_2_ dsa. Nonetheless, the zinc electrode corrodes rapidly and its redeposition competes with the desired cathode dehydroxylation of the glyceraldehyde intermediate. As with RuO_2_ dsa considerable 1,3-PD selectivities were obtained with platinum in neutral media (Fig. S5 and S6†). Glycerol electrolysis at pH 1 was also investigated using platinum anode with Pb and Zn cathode. As shown in Fig. 5 and 6, Pt *vs.* Pb exhibited greater selectivity to 1,3-PD in NaCl, while Pt *vs.* Zn is more suited to 1,3-PD selectivity in KCl. The proposed scheme of anticipated series of reaction steps of the glycerol electrolysis at pH 1 towards the desired selective glycerol-to-1,3-PD conversion is represented in Scheme 3. Although the dynamics of reactions at the anodic component of the electrolysis is well anticipated, the dynamics of reactions at the cathodic component of the electrolysis is equally not simple. Hence, detailed understanding of the results presented in Fig. 5 and 6 is not clear at the moment. However, effect of pH change at near surface of the cathodes is an important factor. Effect of local pH changes at the surface of electrodes during electrolysis that are accompanied by hydrogen or oxygen evolution had been advocated.^41^ Youngkook and Koper used the effect of local pH changes at the electrode surface to explain the mechanism of cathodic reduction of glucose on metal electrodes in Na_2_SO_4_ solution.^42^ Kwon and Koper advanced that hydrogen evolution *via* the reaction: 1/2H_2_O + 2e → 1/2H_2_ + OH^−^, increases the local surface cathode pH. The authors added that surface hydroxide ions promoted isomerisation of glucose from the cyclic (inactive) form to the open chain structures (aldehyde and enol) that are active towards cathodic transformations.

**Scheme 4:**
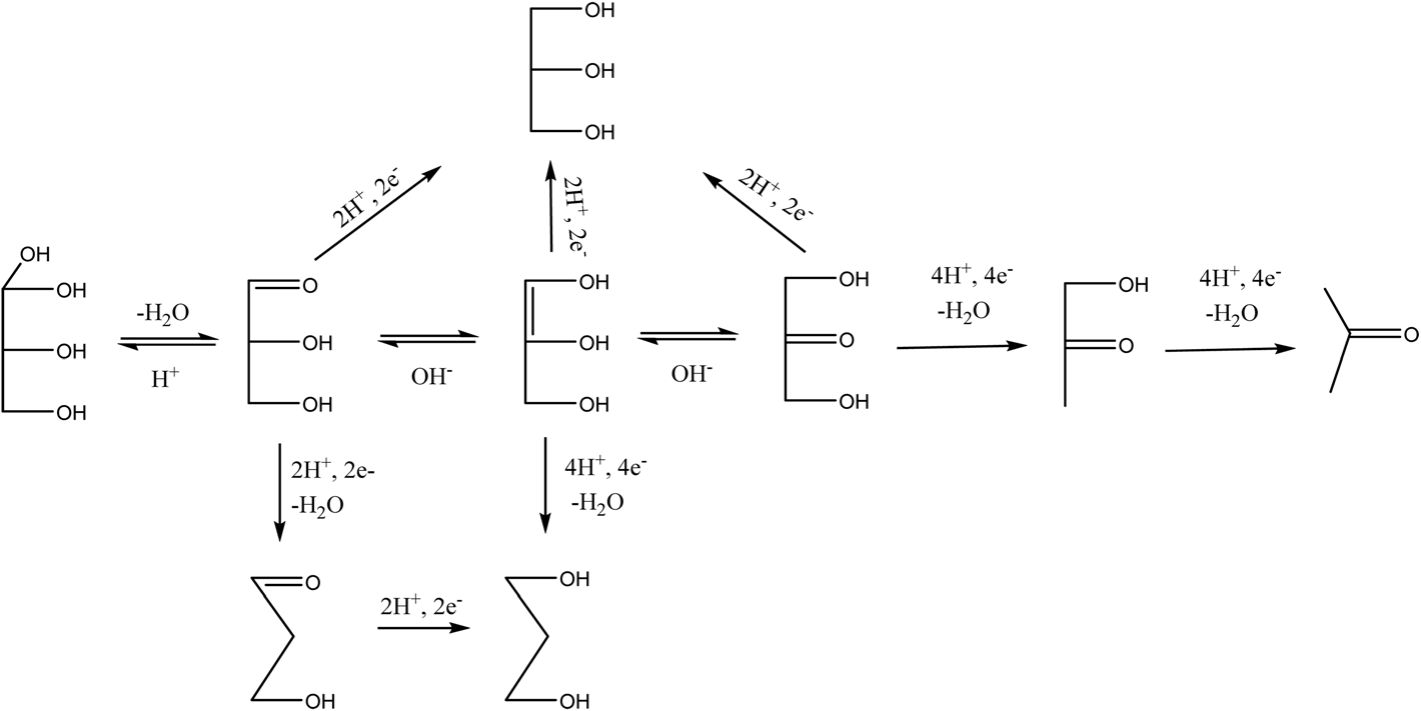
Proposed mechanism of cathodic transformation of glyceraldehyde.

A similar electrode local pH changes effect may be invoked to explain cathodic transformations of glyceraldehyde intermediate media aspect of the present glycerol electrolysis in chloride media. But with the addition, that glyceraldehyde exists in solution as equilibrium between hydrated (inactive) form and free (active) form. Driving the equilibrium towards the cathodic active form involves dehydration which is traditionally an acid catalysed transformation. Cathodic hydrogen evolution causes the increase of the local pH at the cathode surface. The local high pH condition promotes another equilibrium loop in the reaction media between glyceraldehyde, DHA and an enol intermediate (Scheme 4). Free glyceraldehyde and its enol forms are dehydroxylated to 1,3-PD or reduced back to glycerol. Low 1,3-PD selectivities obtained in the glycerol electrolyses in HCl are indication to importance of local high pH condition to achieving high 1,3-PD selectivity. Also it suggests that the enol form is cathodically more active than the free glyceraldehyde form. At the same time the tendency towards full isomerisation to DHA creates a drain on 1,3-PD selectivity.

**Fig. 7:**
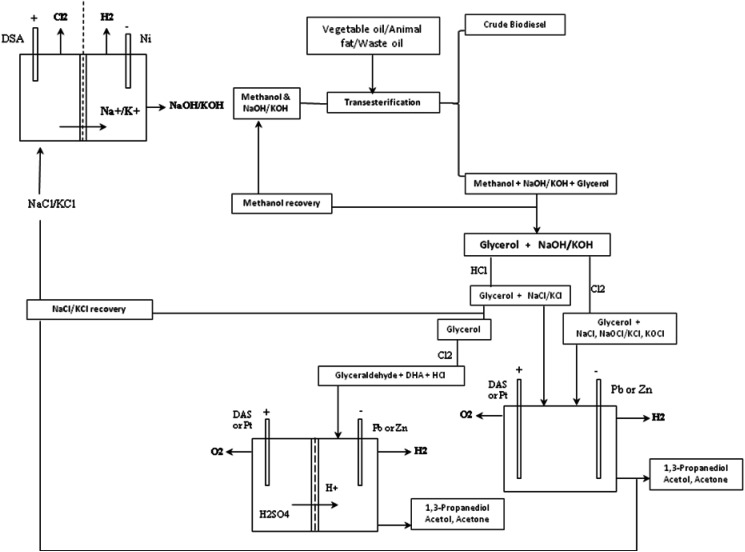
Schematic representation of potential process loop connecting chlor-alkali cell, biodiesel synthesis and glycerol-to-1,3-PD electrolysis.

While low pH conditions favour glycerol-to-glyceraldehyde, the anodic half of the glycerol electrolysis, subsequent glyceraldehyde-to-1,3-PD transformation requires a subtle local cathodic acid-base equilibrium. It appears a compromise condition is achieved at pH 1. It is interesting that the compromise may allows for direct usage of crude glycerol from biodiesel synthesis as cheap feedstock for a commercial electrochemical glycerol-to-1,3-PD process. Most commercial biodiesel production uses alkalis (NaOH, KOH, which are produced by chlor-alkali process), as catalysts. The crude glycerol byproduct of the biodiesel process contains: the alkali catalyst, methanol and other coproducts such as diglycerides and soap. Acidification of the crude glycerol to pH 1 will bring about precipitation of free fatty acid/soap and diglycerides which can allow for the separation of these impurities by decantation. The resulting glycerol still contains methanol, which may be recovered by distillation. The glycerol will require further purification steps to obtain cosmetic or pharmaceutical grade glycerol. However, this partly refined glycerol is suitable for use in the proposed electrochemical glycerol-to-1,3-propandiol process in this study. Also, most chlor-alkali installations use dimensionally stable anodes. One of the electrodes investigated in this study. If a biodiesel production plant is sited near a chlor-alkali installations, it is possible that the proposed glycerol-to-1,3-PD may be able to share facilities with chlor-alkali installations. Thus like in the petrochemical industry, a network loop between chlor-alkali plant and biodiesel plant can be envisaged (Fig. 7). Thus, the glycerol-to-1,3-PD process can be integrated into an existing chlor-alkali plant, thereby representing a sustainable and green route to 1,3-PD.

## 4 Conclusion

We have examined glycerol electrolysis in chloride media. Selective glycerol-to-1,3-PD transformation requires a selective glycerol-to-glyceraldehyde anodic half reaction coupled to a subsequent selective to glyceraldehyde-to-1,3-PD cathodic half reaction. RuO_2_-based dsa and platinum satisfied the glyceraldehyde selectivity and the stability in chloride as selective electrode for the anodic half of the electrolysis. Lead and zinc also displayed cathodic dehydroxylation activity of the hydroxycarbonyl intermediates. Although high glyceraldehyde selectivity was obtained in HCl, it is unsuitable for the cathodic local acid–base dynamics. The purely acidic medium gave high glycerol conversion and low 1,3-PD selectivity, while high 1,3-PD selectivity was obtained in the neutral chloride media but at low glycerol conversion. An optimized conversion and selectivity was obtained in acidified chloride media (pH 1), which may allow for direct usage of crude glycerin from biodiesel synthesis for the electrolysis. This study showed that glycerol electrolysis in chloride media is a potential green and sustainable industrial route to 1,3-PD production.

## Conflicts of interest

There are no conflicts to declare.

## Supplementary Material

RA-008-C8RA00711J-s001
