# Supplementary material for: Towards selective electrochemical conversion of glycerol to 1,3-propanediol
Source: RSC Adv. 2018 Mar 19;8(20):10818–27. doi: 10.1039/c8ra00711j (PMC9078992; doi:10.1039/c8ra00711j)
Supplement: RA-008-C8RA00711J-s001 [file RA-008-C8RA00711J-s001.pdf]

Electronic Supplementary Material (ESI) for RSC Advances.
This journal is © The Royal Society of Chemistry 2018

Supplementary information

Towards selective electrochemical conversion of glycerol to 1,3-Propanediol

Olusola O. James,[1,2] Waldemar Sauter,[1] Uwe Schröder[1]

[1] Institute of Environmental and Sustainable Chemistry, Technical University, Braunschweig, Hangenring 30, 38106, Braunschweig, Germany

[2]Chemistry Unit, Kwara State University, Malete, P.M.B. 1530, Ilorin, Nigeria

Cathode	Mass (g) before electrolysis	Mass(g) after electrolysis	% Mass difference
Pb	9.04190	9.0557	+ 0.15 %
Zn	3.60605	3.3248	- 7.8%

Table S1a: Masses of Zn and Pb as working electrode before and after electrolysis 0.25M Glycerol in an undivided cell with Ti-RuO_2 counter electrode in 0.5M HCl solution (potential -1.8V Sat'd AgCl/Cl, temperature 25 ^oC)

Cathode	Conc (mg/L)	Conc (mg/L)
Pb	0.029	406
Zn	0.045	6630

Table S1b: ICP-OES analysis of the cell content before and after electrolysis reaction

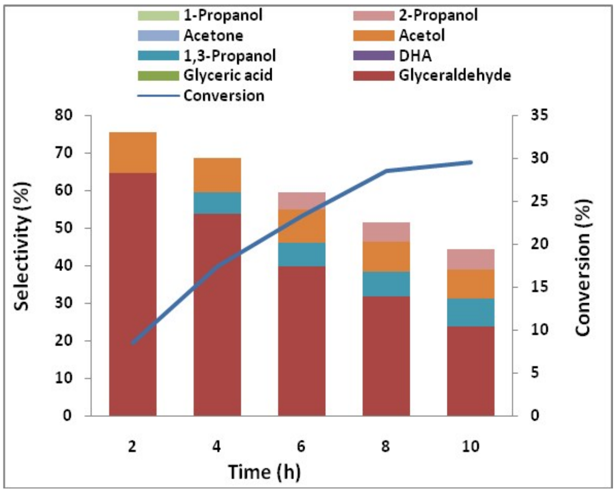

NaCl

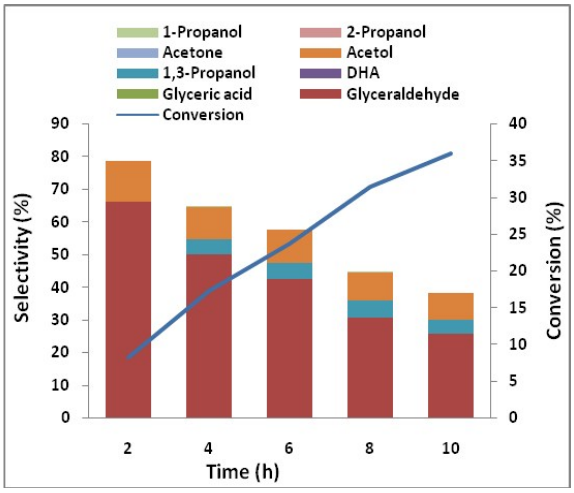

KCl

Figure S2: Conversion and selectivity of oxidation of 0.25M Glycerol in a undivided cell using Pb working electrode and Ti-RuO_2 counter electrode in using 0.5M chloride solutions acidified with HCl to pH 1 (potential -1.8V Saturated AgCl/Cl, temperature 25 ^{o}C)

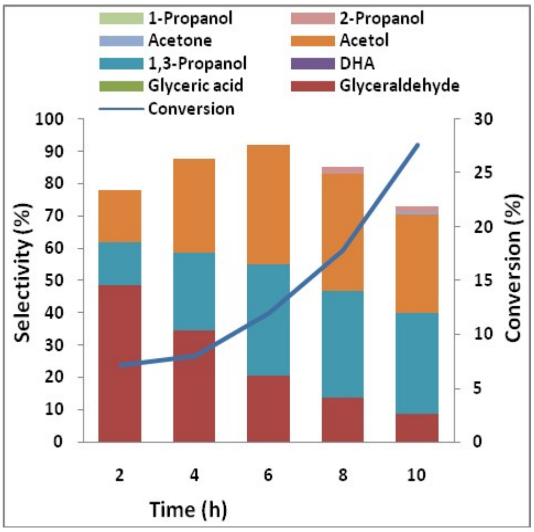

NaCl

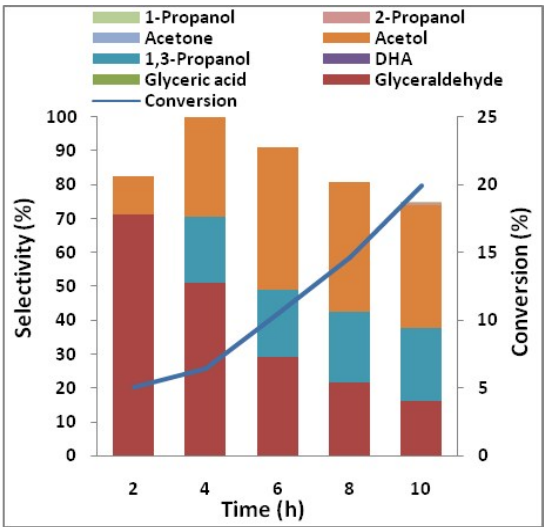

KCl

Figure S3: Conversion and selectivity of oxidation of 0.25M Glycerol in a undivided cell using Zn working electrode and Ti-RuO_2 counter electrode in using 0.5M chloride solutions acidified with HCl to pH 1 (potential -1.8V Saturated AgCl/Cl, temperature 25 ^{o}C)

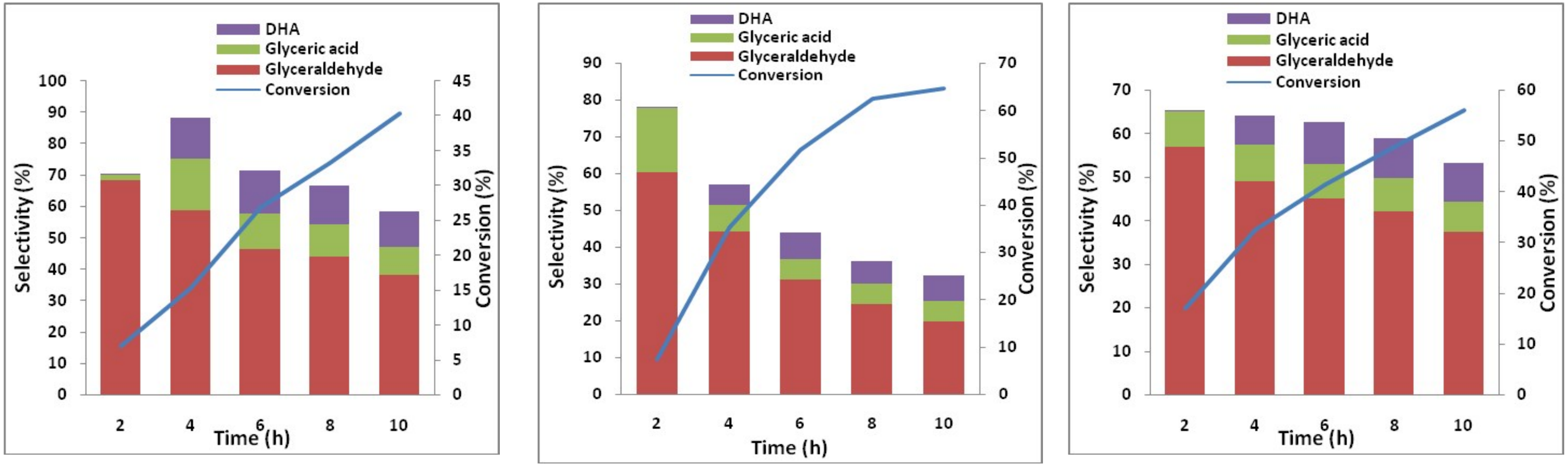

NaCl KCl HCl

Figure S4: Conversion and selectivity of oxidation of 0.25M Glycerol in a divided cell using Pt working electrode and Pt counter electrode in using 0.5M chloride solutions (potential 2.5V Saturated AgCl/Cl, temperature 25 ^{o}C)

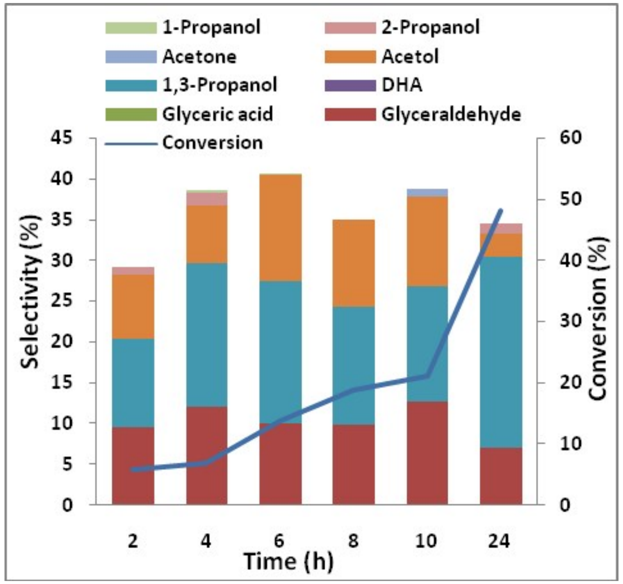

NaCl

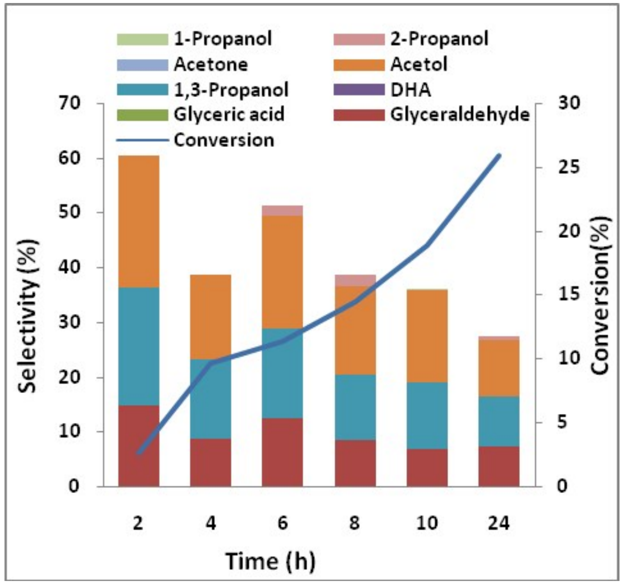

KCl

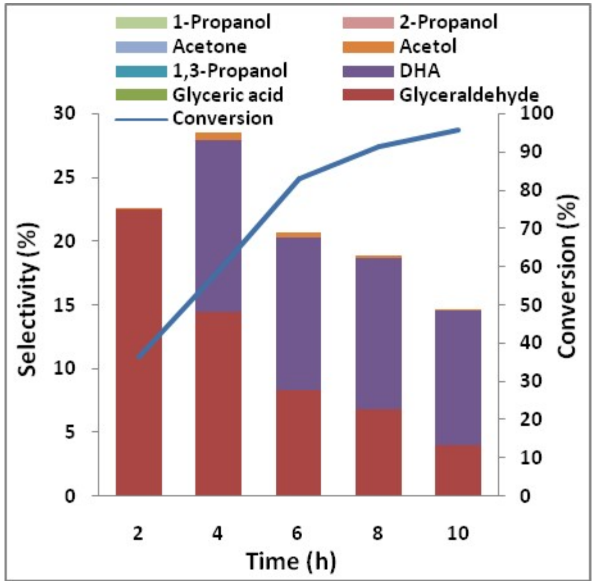

HCl

Figure S5: Conversion and selectivity of oxidation of 0.25M Glycerol in a undivided cell using Pb working electrode and Pt counter electrode in using 0.5M chloride solutions (potential -1.8V Saturated AgCl/Cl, temperature 25 ^{o}C)

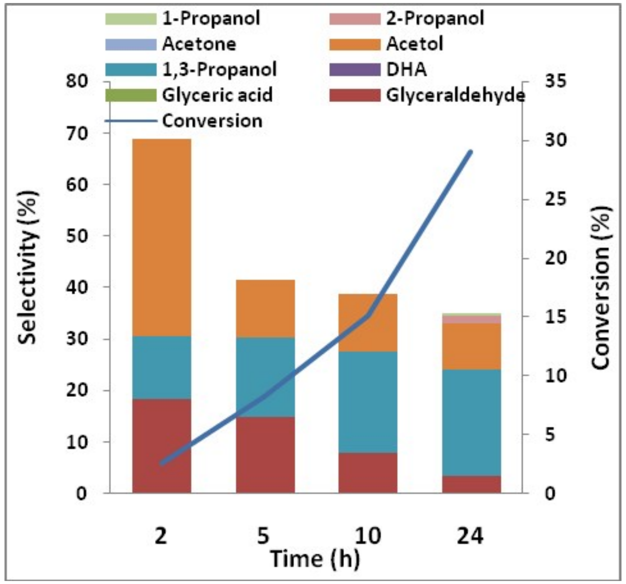

NaCl

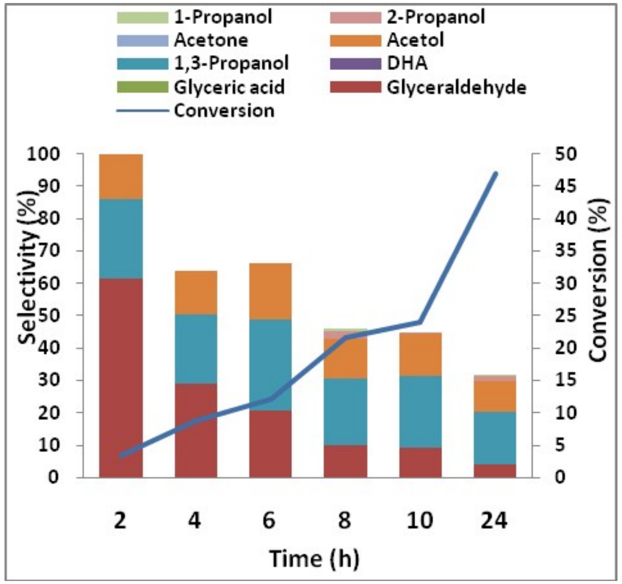

KCl

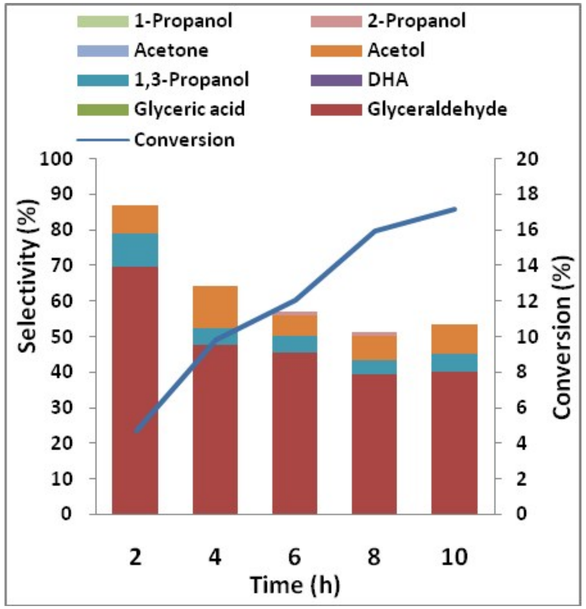

HCl

Figure S6: Conversion and selectivity of oxidation of 0.25M Glycerol in a undivided cell using Zn working electrode and Pt counter electrode in using 0.5M chloride solutions (potential -1.8V Saturated AgCl/Cl, temperature 25 ^{o}C)
